# On the Relatedness and Nestedness of Constraints

**DOI:** 10.1186/s40798-019-0178-z

**Published:** 2019-02-11

**Authors:** Natàlia Balagué, Rafel Pol, Carlota Torrents, Angel Ric, Robert Hristovski

**Affiliations:** 1 Complex Systems in Sport Research Group, Institut Nacional d’Educació Física de Catalunya (INEFC), University of Barcelona (UB), Av. de l’Estadi, 12-22, 08038 Barcelona, Spain; 2 Real Federación Española de Fútbol (Spain), Complex Systems in Sport Research Group, Institut Nacional d’Educació Física de Catalunya (INEFC), University of Lleida (UdL), Complex de la Caparrella, s/n, 25192 Lleida, Spain; 3 Complex Systems in Sport Research Group, Institut Nacional d’Educació Física de Catalunya (INEFC), University of Lleida (UdL), Complex de la Caparrella, s/n, 25192 Lleida, Spain; 4 F.C. Barcelona, Barcelona (Spain), Complex Systems in Sport Research Group, Institut Nacional d’Educació Física de Catalunya (INEFC), University of Lleida (UdL), Complex de la Caparrella, s/n, 25192 Lleida, Spain; 5 Complex Systems in Sport Research Group, Faculty of Physical Education, Sport and Health, Ss. Cyril and Methodius University, Dimche Mirchev, Skopje, 1000 Republic of Macedonia

**Keywords:** Task constraints, Perceived affordances, Constraints-led approach, Nested organization, Timescales, Circular causality, Fast-changing constraints, Slow-changing constraints

## Abstract

The purpose of this opinion paper is providing a platform for explaining and discussing the relatedness and nestedness of constraints on the basis of four claims: (a) task constraints are distributed between the person and the environment and hence are relational variables, (b) being relational, task constraints are also emergent properties of the organism/environment system, (c) constraints are nested in timescales, and (d) a vast set of constraints are correlated through circular causality. Theoretical implications for improving the understanding of the constraints-led approach and practical applications for enhancing the manipulation of constraints in learning and training settings are proposed.

## Key Points

- Constraints are interdependent entities acting at different timescales.
- Task and task constraints, distributed between the person and the environment, are emergent properties of the organism/environment system.
- The knowledge of the hypothesized temporally nested organization of all types of constraints may provide a basis for improving the understanding and efficiency of learning/training processes.

## Introduction

Constraints determine the way in which the multiple components of complex systems self-organize [1, 2] to produce reliable macroscopic functions [3]. According to Kugler et al. and Pattee, contrary to dynamic laws which are incorporeal and universal, constraints are always physically embodied and local [3, 4].

The concept of constraints is used in different scientific fields (e.g., mathematics, physics, computer science, biology, and linguistics) and refers to boundary conditions, limitations, or design features that apply restrictions to the degrees of freedom of a system, thereby indicating the trajectories that the system may exhibit [3]. Constraints-led approaches based on Newell’s model [5]have been applied to numerous movement science and sporting fields in recent years, including skill acquisition [6, 7], motor development [8], motor performance [9], medicine [10, 11], physical therapy and rehabilitation [12–14], physical conditioning [15, 16], sports biomechanics [17, 18], creative behavior [19, 20], and sport injuries [21]. Due to the integration of variables studied in different disciplines such as physiology, biomechanics, and psychology, the constraints-led approach has been suggested as a possible unifying framework for sport performance studies [22]. Although the proposal has received criticism [23–26], the integrative and practical potential of the constraints-led approach is indubitable.

Newell’s classification [5] distinguishes three categories of constraints: organismic, environmental, and task-related. Organismic constraints are related to personal characteristics and are classified as structural or functional. The so-called structural organismic constraints tend to remain relatively constant over time (anthropometric characteristics, body composition, muscle architecture, and typology or personality), compared to “functional” organismic constraints, which change at a faster rate (physical condition, fatigue, motivation, cognition, effort perception, heart rate, or lactate concentration). Environmental constraints are external to the organism and were initially distinguished as general (e.g., climate, temperature, light) and task-specific (e.g., implements, apparatus) [5]. As this distinction was not considered clear enough, Newell’s initial proposal evolved towards the current classification which considers as environmental constraints all those outside the person, including the implements or apparatus, which were initially classified as task constraints [27]. Additionally, both physical and sociocultural constraints (e.g., fans’ support, social pressure, score) are treated as environmental constraints [28].

Task constraints are usually defined as those specified by the task to be performed(e.g., ball size and shape, specific goals to be achieved, boundary lines, playing field length, number of opponents and teammates involved, situational characteristics of opponents such as players’ relative position, and approach speed) [29]. They are related to the task goal, the environmental information, and the instructions and rules. Rules and instructions can simply constrain the task (e.g., say what is forbidden) or specify the response dynamics (e.g., prescribe the action solution or the pattern of coordination). For instance, a referee can award a penalty kick and signal the kick but does not impose the task solutions, i.e., actions (direction of the kick, type of shoot, etc.). In contrast, a competition rule may require the performance of a set of gymnastics skills. Thus, task constraints can be divided into specific, when they specify the movement form or action to be performed, and non-specific, when they do not specify it [5].

The ecological dynamics classifies task constraints as being instructional (rules and instructions), and informational, that is, related to the visual, acoustic, and haptic information that can be directly perceived by the performer, which is the basis of “affordances” (opportunities of action) [30]. While affordances have been recognized as relational [31–33], generally, task constraints have not yet been acknowledged as such. We discuss here how all types of tasks and task constraints, not only affordances, are distributed between the performer and the environment and are emergent properties of the performer–environment system.

In a similar vein, although some authors have referred to the timescales of task constraints [17, 34–36] and their nested organization [37], most of the previous work on the topic has focused on the behavioral space-time dynamics and space-time task constraints, as well as on the circular causality between the components and collective levels that form the behavioral variables [29, 38]. We plan to focus here on constraints, and not on behavioral variables, and explain how all types, not only task constraints, are interdependent, correlated through circular causality, and organized in a nested, i.e., embedded fashion, at levels defined by their characteristic timescales. Although behavioral variables (e.g., opponent’s actions) may act also as constraints, in research and practice, one should always distinguish the role played by each variable in the model. In this respect, opponent’s actions play the role of constraints when studying the game dynamics of one team and the role of state variable when studying the game dynamics of their opponents.

This opinion paper explains and discusses the relatedness and nestedness of constraints on the basis of four claims: (a) task constraints are distributed between the person and the environment, (b) task constraints are emergent entities, (c) constraints are nested in timescales, and (d) constraints are correlated through circular causality. Additionally, some theoretical and practical implications, addressed to improve the understanding and effectivity of constraints manipulation in learning and training settings, are proposed.

## Why Task Constraints Are Distributed Between the Person and the Environment

**Fig. 1:**
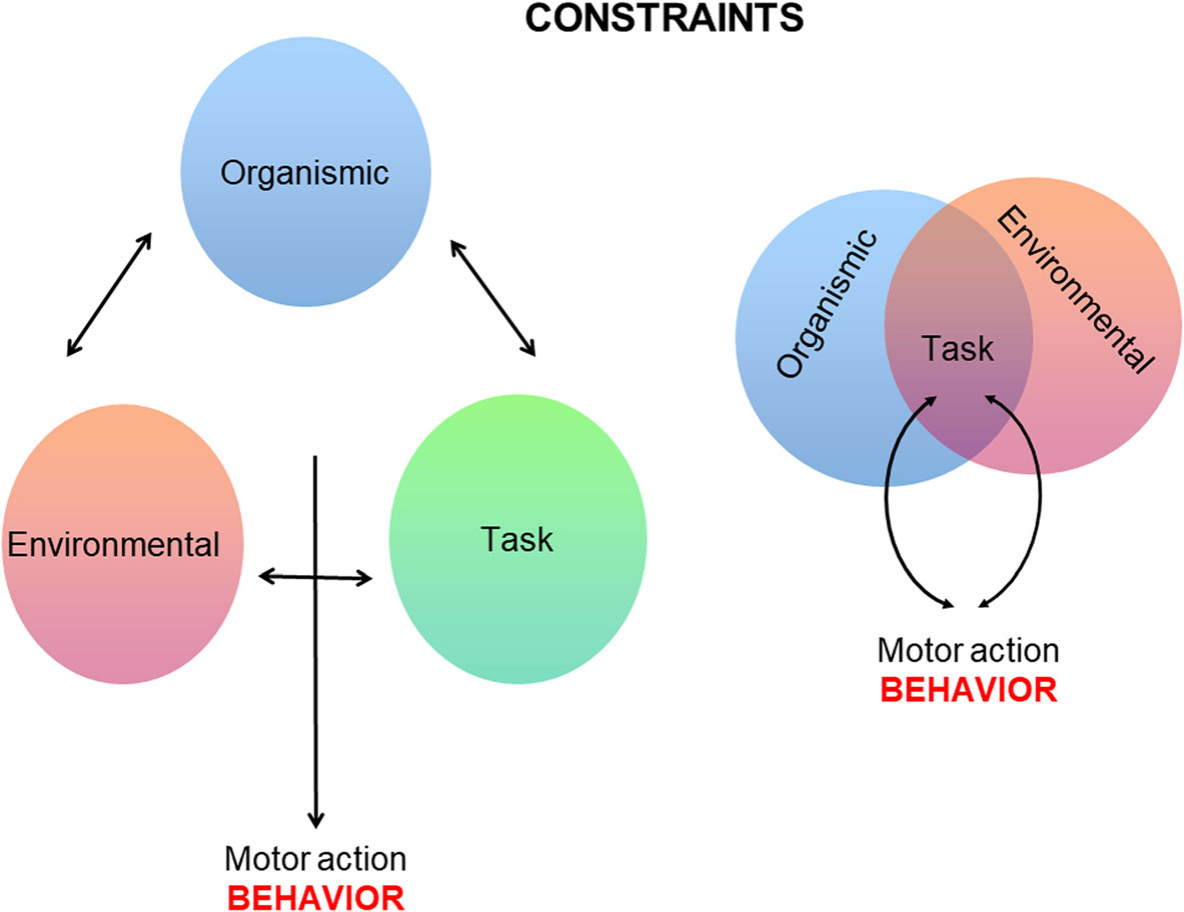
Left: Newell’s model [5]; organismic, environmental, and task constraints as independently defined interacting entities. Right: organismic and environmental constraints as independently defined interacting entities, and task constraints as emergent properties of the organism–environment system

We claim that task constraints, unlike the other two sources of constraints, are distributed variables and can only be defined at the systemic organism/environment level. It is worth noting that we consider organismic constraints, other than morphological, as dispositional properties that mold the establishment of functional relationships with the environment [39]. Disposition is a tendency, liability, or proneness to act or react, or fail to act or react, in a certain way in certain circumstances [40] (Fig. 1).

The objects of the environment and their properties (e.g., a ball and its size) *become task constraints* only when interacting with or relating to a goal-directed purposeful organism. Without an organism seeking for its goals, physical properties of the environment are just that - physical properties of nature. A ball may constrain other balls physically (e.g., gravitationally or by forces of friction) in the store, but such constraints cannot be called task constraints, simply because tasks can be defined only in the relation between goal-directed organisms and their environments. In general, the term constraint is by definition relational, because one can always ask “what constrains what.” Even if a physical object, or an organism, constrains itself, that very constraint is a relation of the object or organism with itself. Without that relation, the term “task constraint” loses its meaning, and it only exists as a mere environmental property. As environmental *properties* become *task constraints* only *for* a certain organism, if the organism–environment relation vanishes or changes, the task *inevitably* vanishes or changes too. Designing a task means designing a certain *relation* between the performer and the environment, outside of that relation a task does not exist.

The organism–environment interaction is defined as an influence of certain environmental properties on a goal-directed entity (organism) and sometimes vice versa. For example, the ball trajectory influences the actions of the player but also the player may change the ball trajectory (two-way interaction). On the other hand, the ball size and weight influence the perception–action of the player but not vice versa. It is not a two-way interaction. In both cases, if the goal-directed organism is not involved, we can say that environmental properties are not task constraints because we take the organism out of the equation. Any information, object, or force may potentially act as a constraint, but at each moment, only a subset of constraints acts significantly on the system (performer or team).

Whereas organismic constraints simultaneously belong to the organism *and* to the organism–environment system, but *not* to the environment *alone*, and the environmental constraints belong to the environment *and* the organism–environment system, but *not* to the organism *alone*, tasks and the associated *full set* of task constraints are distributed within the organism–environment system and, *as a set*, do not belong neither to the organism nor to the environment *alone*. For example, the height, the strength, the readiness to act, the attentional focus, and the task goal are organismic constraints, but not environmental constraints. The ball size and weight are environmental constraints, but not organismic constraints. The full set of task constraints, on the contrary, is a *union* of both, the organismic (task goal) and the environmental constraints. As they are distributed and form a relationship at the level of organism–environment system, they can only be defined at systemic level. This is why task constraints differ ontologically from organismic and environmental constraints.

The inseparability of the organism–environment system [41] itself means that tasks, and hence task constraints, cannot be defined as a third separate entity that merely interacts with the environmental and organismic constraints. If the organism–environment system is the union of the elements of the organism, the environment, and the organism–environment system, then by definition, there can be nothing outside of this system (such as tasks or task constraints) which would interact with this system or its subsystems. Hence, in the Venn diagram, task constraints are represented as intersection of the organism–environment system, just as would follow from Turvey [41] (see Fig. 1, right). It should be noted that although some authors have used the intersection of circles to represent the interactions of the three different and independently defined types of constraints of Newell’s model [22], in Fig. 1 (right), the intersection represents the distributedness, relatedness, and emergent nature of task constraints.

### Affordances as Informational Task Constraints

Gibson [32] postulated that humans can perceive the features of the environment as possibilities for action and defined the relation of perception and action in terms of a circular flow. According to the perception–action cycle, the environment is not perceived in terms of its objective properties (distances, angles, etc.) or in terms of expectations and mental representations linked to performance solutions [42]. The properties of the environment are scaled to the motor abilities of the performer [43], i.e., the environment is perceived in terms of what the organism can do with and in it, that is, in terms of affordances. In other words, affordances are values of use of objects or surfaces.

**Fig. 2:**
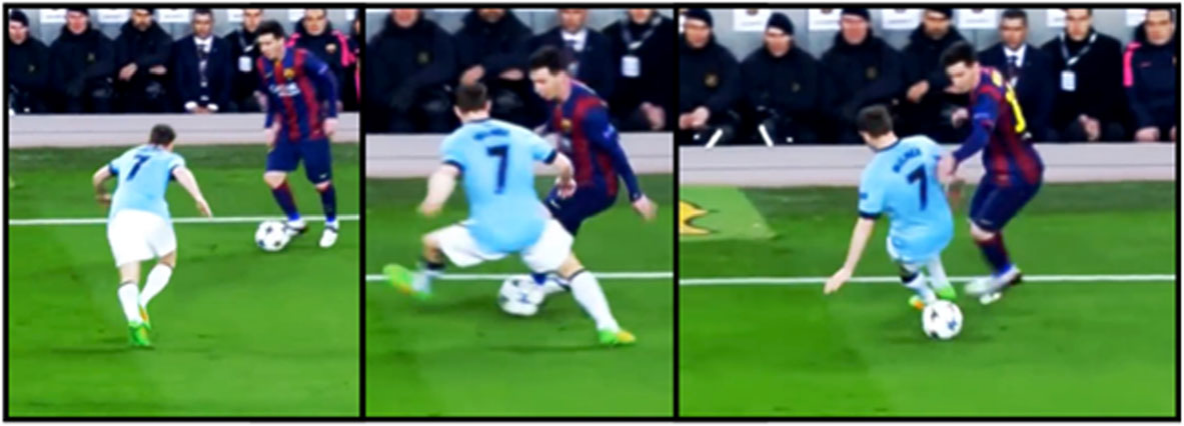
L. Messi enacting a tunnel to escape from a defender close to the touchline

Through acting in the environment, the performer perceives such affordances; thus, it is the interaction of the organism with information from the environment that creates the informational constraints which define the affordances [44]. Figure 2 shows an example of affordances during a soccer match. Near the touchline, the player possessing the ball has reduced possibilities for escaping from the defender, who takes the opportunity to press forward. L. Messi, the attacker, perceives (in a few tenths of a second) the affordance of escaping from his defender by performing a tunnel. For Messi, this environmental property emerges and vanishes in a fraction of second, and hence, the *perception* of the affordance emerges and decays at the same timescale. Organismic constraints like speed of movement, strength, motor abilities, level of fatigue, motivation, or values (e.g., fair play), among others, constrain the affordances used by players during the match. It is important to point out that Messi’s goal was probably to escape from the defender and maintain the possession of the ball, but not specifically by performing a tunnel. However, his goal constrained his attention and his attention constrained his perception, as will be explained below (see Fig. 4). Thus, the tunnel affordance, like other action solutions in sport that cannot be planned in advance, emerges spontaneously from the performer–environment interaction. Player’s interpersonal distances, the distance between feet, or the players’ relative velocity become task constraints only when they are actively perceived by performers; therefore, it can be said that informational task constraints are distributed between the organism and the environment.

### Instructional Task Constraints (Rules and Instructions)

Instructional constraints are directly related to the task goal or action solution. They can be specific and provide information on how to perform the action, or they can be non-specific (e.g., instruct what to avoid instead of what should be done) [45].

Rules and instructions may be considered as environmental information provided via social systems and transmitted through language (e.g., coach instructions, training/competition rules). This type of environmental social information should be assimilated by the performer in order to become a task constraint [46–48]. In fact, this information cannot be defined without goal-directed organisms for which those rules and instructions are valid. It is important to note that instructions, themselves, are just third person (e.g., coach’s, referee’s) references for the preferred in situ relations between the performer and the environment. This is one of the reasons why instructions do not have the same effects on all instructed performers. This means that goals, rules, and instructions, as other task constraints, are relational and distributed variables which exist at the systemic organism/environment level.

## Tasks and Task Constraints Are Emergent Properties of the Organism–Environment System

Tasks are understood here as a set of interacting task constraints. As tasks and task constraints are distributed between the organism and the environment, they are necessarily emergent, either by design (e.g., through instructions) or spontaneously, i.e., by self-organization (e.g., Fig. 2). Properties that exist only at systemic (e.g., organism–environment) level, and not at levels below (e.g., organism or environment alone), are called emergent properties [49, 50]. In other words, for a property to be emergent, the necessary and sufficient condition is not to be a property of the system components. Note that this definition does not pose additional criteria to the system components properties and their interactions. Then, it is obviously incidental and not essential to the definition if the component interactions are designed, prescribed, or arise spontaneously, whether the system has central or distributed control or if the components have (or do not) a representation of the global system behavior. The concept of emergent property has the same meaning for technical (e.g., robots), biological, physical, or social systems. However, it is important to note that not all properties at systemic (macro) level are emergent. For example, the mass of a system is only an extensive property because its components have the property of mass themselves.

While in physical, chemical, and biological systems emergent properties arise dominantly through self-organization, in social systems, the interactions among components (e.g., players) are sometimes planned, prescribed, and designed by an external agent (e.g., a coach). On the other hand, e.g., in small-sided games and matches, there are emergent properties which arise spontaneously by self-organization. This is because a large set of constraints and interactions between players change spontaneously, that is, they are not specifically designed or prescribed by the coach. When these interactions change, the task changes as well. Thus, during matches, old task constraints decay and new task constraints arise. In this case, one can say that tasks self-design.

Task solutions, i.e., actions, always emerge from the in situ interactions between the organismic constraints (e.g., level of stress, fatigue or strength of the performer) and the environmental constraints (e.g., opponent’s behavior, terrain). Additionally, actions emerge from the interaction between many other microscopic degrees of freedom acting at lower levels (nervous system, muscles, tendons, bones, joints, etc.).

Task constraints may have non-linear or non-proportional effects on performer’s actions. This means that while a change in a set of task constraints may have no visible effects, a further small change may produce a qualitative reorganization of the whole system [45]. For instance, while a substantial increase in the time on task may be adequately compensated through psychobiological synergies, an additional small increase in exercising time can suddenly produce task disengagement due to exhaustion [51], or a small deviation of the ball trajectory during a soccer match can lead to ball recovery and complete re-organization of both teams (e.g., during a counterattack). Game dynamics, characterized by its transitions, changes in ball possession, space occupation, tactical patterns, play rhythm, etc., may sometimes be guided by these non-linear effects which greatly increase the uncertainty of the game. These sudden changes, products of the interactions between a set of task constraints, emerge as *new tasks* spontaneously via self-organization (i.e., without being previously designed or imposed on the players or teams).

## Constraints Act at Different Timescales

Some constraints change slowly with respect to the macroscopic function they produce and thus have a long-lasting effect and may be experienced as constant [51]. Newell [5] called them “structural” because they “freeze” the degrees of freedom. We propose calling them “slow-changing constraints” because they change at lower rates than “fast-changing constraints” (called “functional” by Newell). It is important to note that the terms “slow” and “fast” are *relative*. Constraints that are slowly evolving with *respect* to some more rapidly evolving ones can be treated as fast with respect to some variable that evolves over a longer timescale. For instance, the somatotype is a slow-changing constraint and the affordances are fast-changing constraints with respect to player’s technical actions during the game. The rates of change of constraints, having longer- and shorter-lasting effects on behavioral variables, reveal a nested organization of constraints in levels and timescales, which may have relevant implications when planning interventions (see the “Theoretical and practical implications” section).

### Organismic and Environmental Constraints

Organismic constraints evolve structurally and functionally through the interaction with environmental constraints and vice versa. Slow-changing environmental constraints shape slow-changing evolutionary organismic constraints or traits (e.g., human structure and functions); relatively faster changing environmental constraints (e.g., fans’ support) shape faster changing organismic constraints or states (e.g., mood); and even faster changing environmental constraints (e.g., ball trajectory) shape the even faster organismic constraints (e.g., perceptions). In turn, relatively slow-changing organismic constraints, such as habits, affect slow-changing environmental constraints (e.g., microclimate or relief paths), and faster changing organismic constraints (e.g., attention focus) produce faster changing environmental constraints (e.g., ball direction). These are usually two-way interactions, which can be related indirectly (e.g., through actions). Furthermore, constraints acting at different timescales also interact among them through circular causality (see the “Multilevel and nested organization of constraints” and “Correlation of nested constraints through circular causality” sections).

**Fig. 3:**
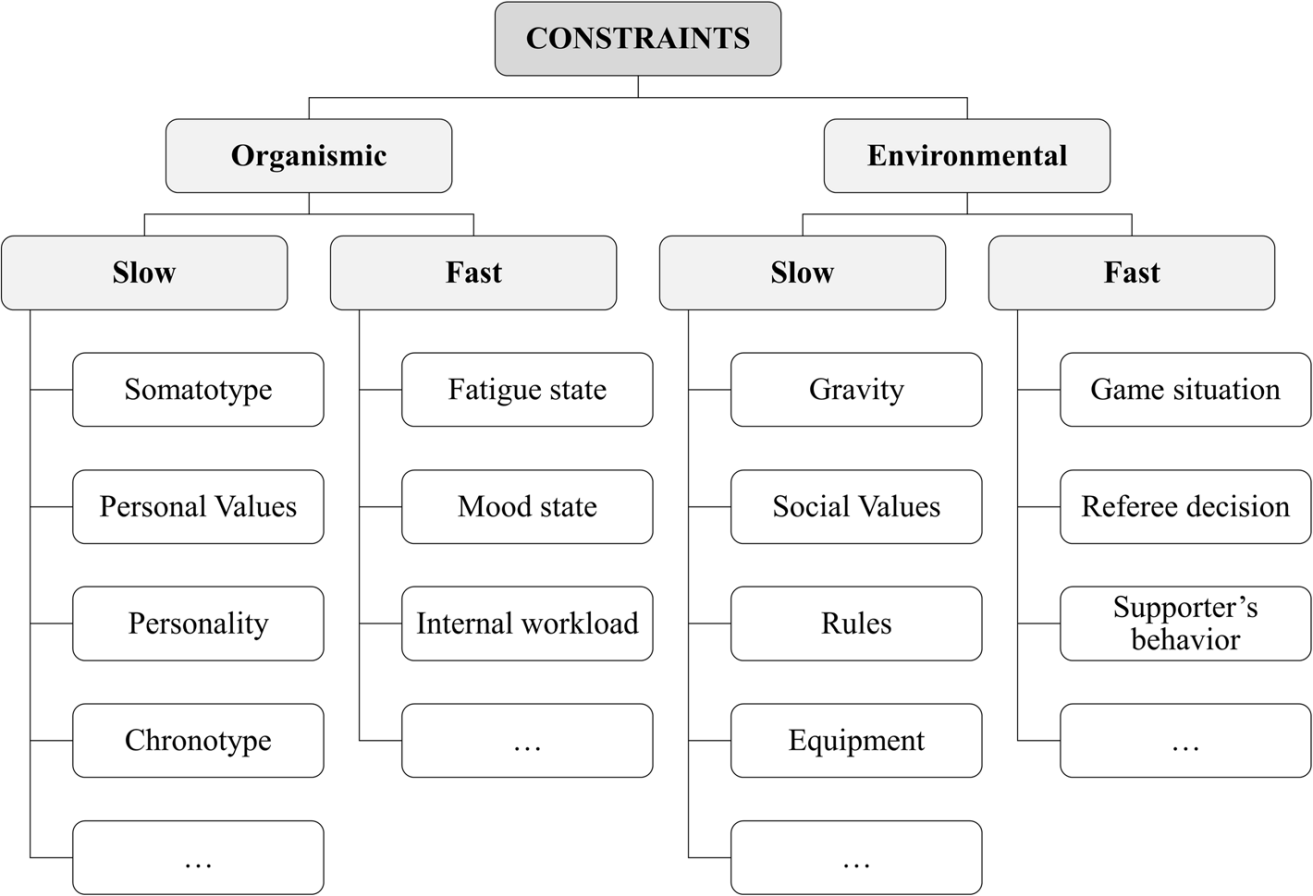
Classification of organismic and environmental constraints according its relatively faster or slower rate of change. Some examples are provided

Figure 3 shows some examples of organismic and environmental constraints with faster and slower rates of change. As a guide, personal values and competition rules may change over decades, fatigue state and supporters’ behavior may change within days or months, and internal workload and game situation may change within seconds or minutes.

### Task Constraints

As the environmental information can either be actively perceived by the performer, e.g., players’ relative position and approach speed [29], or create personal goals and intentions in the performer, task constraints may change at very different timescales. For instance, perception of affordances may occur within fractions of a second, task goals within minutes, team strategies within hours, and competition rules within decades.

## Multilevel and Nested Organization of Constraints

The rate of change of constraints is related to their timescale effects on behavioral variables. The faster a constraint changes, the shorter its effects on the behavioral variable, and vice versa. The different timescales of evolution of organismic, environmental, and developmental (ontogenetic and phylogenetic) constraints were briefly acknowledged in previous research [5, 34]. However, the nested organization of constraints in levels and timescales in human systems has only recently been discussed in the case of task constraints [37]. These authors showed that task constraints on motor behavior are distributed across many interacting time scales rather than being provided at a single common timescale. To date, most of the relevant research has been conducted on the problems of how a single or a couple of (predominantly task) constraints channelize certain behavior within a single time scale. Torrents et al. provided some evidence of multilevel synergic effects between the team and player dynamics when changing task constraints. The exploratory capacity at the team level was significantly lower when professionals played in numerical superiority, and this was compensated by an increase in individual exploration and vice versa [52]. Due to the lack of research on the nested organization of constraints, more studies are needed to assess their multilevel effects on the behavioral dynamics at the level of players, dyads, and teams. At the player level, the teammate anthropometry has been shown to constrain the action of dribbling in 1-on-1 basketball sub-phases [53]. At the dyadic level, the distance to the nearest opponent constrains the pass options [54]. Finally, at the team level, the collective behavior constrains players’ actions [55]. During a game, different solutions emerge from the influence of constraints interacting at different timescales, from short (i.e., fatigue and emotions) to long (i.e., playing style and league culture).

**Fig. 4:**
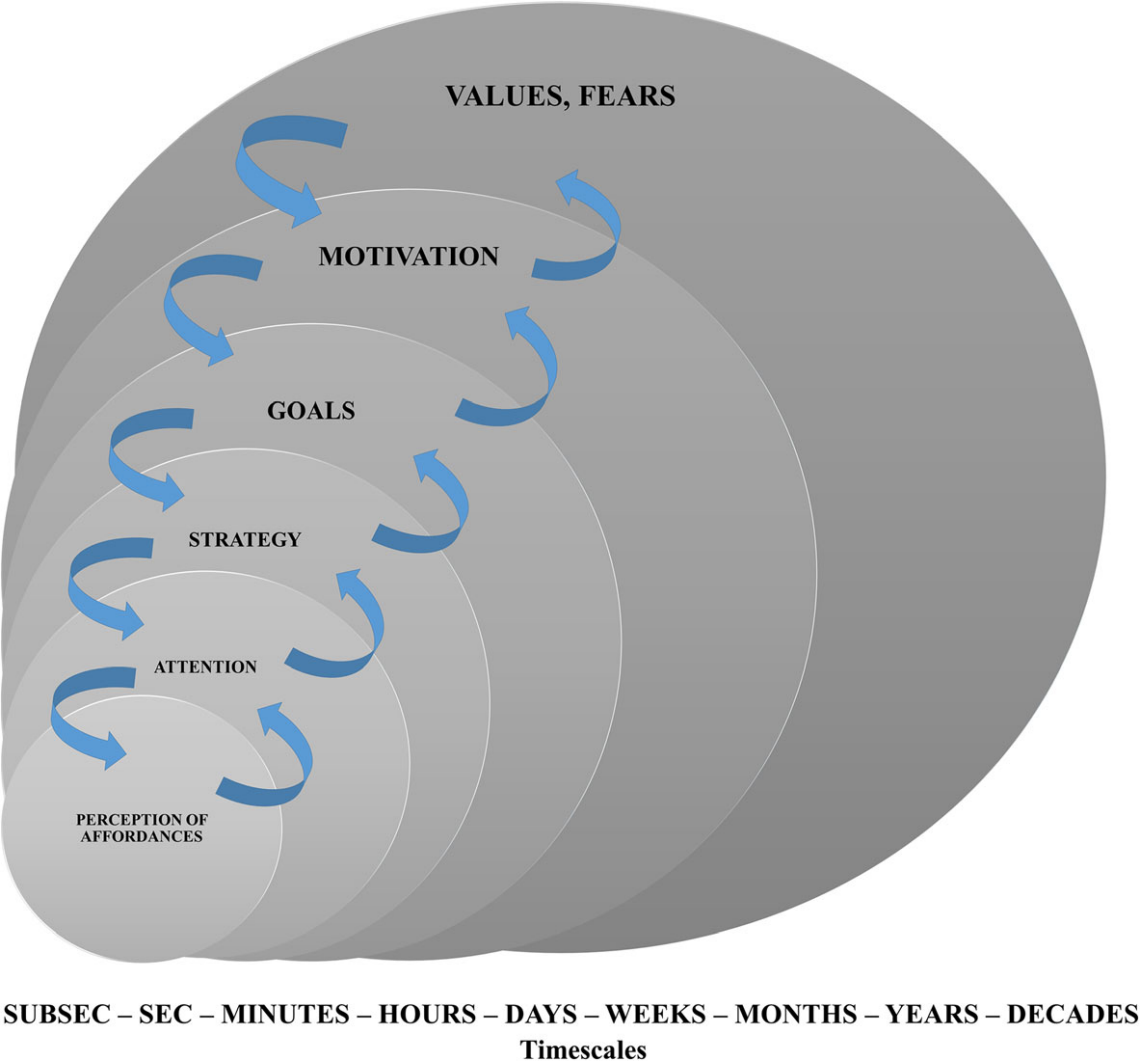
Example of nested constraints operating at different timescales and correlated through circular causality. The exact timescales given in the figure are only orientative (e.g., goals or motivation can be defined at different timescales)

We were not able to find any previous studies discussing the nestedness of the whole set of constraints (organismic, environmental, and task) and their possible circular causality relation. Our claim here is that in sports, all types of constraints, not only task constraints, possess this nested characteristic. Figure 4 shows an example of the multilevel nestedness and correlatedness of constraints. Values (lasting decades) constrain competition motivation which varies over a faster timescale (e.g., weeks, months), which in turn constraints short-term goals (e.g., days or weeks or) and competition strategies (e.g., lasting hours or minutes—a whole match). These constrain the performer’s attention (e.g., minutes, seconds) and, in turn, the perception of his/her affordances (from fractions of a second to seconds), and other muscle processes defined at an even smaller timescale (e.g., metabolic pathways). Relative workloads are a nice example of action-scaled affordances that constrain the metabolic pathways. Short-term goals constrain attention not only through top-down pre-planned strategies. Under a fast-changing constraints regime, as occurs during sporting competitions, goals (e.g., escape from the defender) directly constrain the perceived affordances, as shown in Fig. 2.

The sequence of nested constraints represented in Fig. 4 can, in turn, be reproduced at different timescales. For instance, the goal of having a successful sport career lasts longer than the goal of winning a championship, winning a match, or winning ball possession during the match. We can refer to fatigue status as being acute (days) and recovering fast, or chronic (months) and recovering slowly, or define workloads in the short term (session), mid-term (microcycle), or long term (season). It is worth to point out here that one may find slow-changing constraints evolving over decades not only at social or personal level (e.g., values) but also at molecular level (e.g., epigenetics).

The manipulation of constraints has been widely applied in motor learning and sport training, and specifically in small-sided games [56]. However, due to the limited literature capturing the nested structure of game constraints [57] and the relation between such levels [58], the concept of nested organization of constraints is still under-researched.

## Correlation of Nested Constraints Through Circular Causality

Constraints at upper levels (slow-changing constraints) subjugate those at lower levels (faster changing constraints), which in turn form the constraints at the upper level (circular causality). As many of the levels are related through circular causality (see Fig. 4), the correlation of constraints does not act only from top-down but also from bottom-up, that is, the slowly changing constraints, such as personal values, fears, goals, and motivation levels, create a long-term context impinging on the faster changing variables such as strategy and affordances. On the other hand, fast-changing constraints such as affordances influence the performance level (positively or negatively) and consequently the goal motivation and values. Interventions at the slowly changing constraints level (personal values, fears) enable a supporting context for successful intervention at the rapidly changing constraints level (goals, strategy, affordances) which is a prerequisite for successful behavioral dynamics in sports practice. In turn, a successful intervention at the level of fast-changing constraints (affordances) enhances slowly changing constraints (motivation, goals, and values).

## Theoretical and Practical Implications

The distributedness and emergence of task constraints, as well as the interdependence of constraints and their nested organization in levels and timescales, has some relevant theoretical and practical implications for planning interventions. By defining task constraints as relational and emergent properties, we propose a dimensional reduction of Newell’s model, passing from three different and independently defined types of constraints (organismic, task, and environmental) to two (organismic and environmental), with the task constraints being a systemic property emerging from the interactions between subsets of both (see Fig. 1).

Furthermore, the interdependence and nested organization of constraints offer some practical advantages. An intervention in slow-changing constraints situated at upper levels (e.g., personal values) provokes a correlated cascade of effects on constraints acting at lower levels (i.e., motivational, attentional, conditional, biochemical, etc.). Due to their long-term evolution, upper levels (values, motivation, etc.) provide the general channelizing context for the detailed manipulation of task constraints. If such long-term constraints [59] decay, the whole system of faster constraints decays, and vice versa, and if they enhance, the whole system of faster constraints is enhanced. For instance, if a value such as active sports participation is high and stable, the motivation for practice increases, and thus, the context for manipulating workload properties (volume, intensity, complexity) and learning from affordances enhances as well. Such increase in workloads and fast and accurate perception of affordances increase the likelihood of goal constraints achievement (performance level) and, due to the circular causality, back-propagates enhancing and stabilizing the motivation [60] and value given to sport practice. In contrast, reduced long-term personal values towards sport practice reduces the motivation of athletes for interacting with challenging training/learning environments and produces cascade effects towards slower learning/performance effects (e.g., slower attunement to the environmental information and affordance perception) and general performance stalemate. Through circular causality and back-propagation (i.e., bottom-up effects), this decay in affordances perception may lead to the further decay of motivation and personal valuation of sports activity, which could bring about a nonlinear effect: a drop-off in sports participation. To prevent such drop-off and other nonlinear effects like sports injuries [21], the adequacy of task constraints (i.e., the adequate manipulation of environmental constraints in regard to the individual abilities) is crucial because it may enhance attention, and thus personal goals, motivation, and long-term personal values towards sport practice (see Fig. 4).

The nestedness of constraints can be found in other examples. A player constrained by the fair play value perceives different affordances than one who is not constrained by this fair play (e.g., the first has a vanishingly small likelihood of deliberately kicking the legs of a dangerous football attacker). The fear of failure or fear of success [61], acting as slowly changing long-term constraints, affect competition goals and strategies, attention, perceived affordances, and eventually, performance. By manipulating the number of players, the size of the pitch, the score, or some playing/training strategies, coaches channelize all levels down, i.e., manipulate faster changing constraints(from tactical to biochemical), in a correlated way. Under this perspective, proposing, for instance, tasks detached from the game to activate specific metabolic pathways (e.g., aerobic/anaerobic) loose sense because the physiological/biochemicalactivation arises as a consequence of the nestedness of constraints when players respond to task features.

Coach instructions, as an environmental constraint, should be mainly addressed to processes developed over longer timescales, e.g., values, goals, and strategy. Instructions imposing specific action solutions (e.g., related to technical skills like dribble, pass, shoot, etc.), which may change over very short timescales during a game, can compete with the actively perceived affordances of the players and be counterproductive [62]. Thus, the coach type of instruction should be adequate to the action timescale and fit with the performer’s organismic constraints. Additionally, differences in physical condition, expertise, level of skills, fatigue, or emotional state can change the perceived affordances of the players/athletes and decrease the effectiveness of some instructions. In turn, personal differences in cognitive abilities and motivational drivers can also produce changes in the effectiveness of instructions. While a motivated athlete can transform coach instructions in personal goals, a demotivated athlete may not. Whereas for actions requiring longer timescales (e.g., strategic planning), information via language may be effective in motivated and cognitively attuned athletes, for actions requiring shorter timescales (see Fig. 2), information coming from other perceptual systems should prevail.

Finally, the correlation of goals, intentions, and strategies at different timescales (e.g., short-, mid-, and long-term goals) seems crucial for long-lasting performance results, either defined at individual or team level.

## Conclusions

In this opinion paper, we explain and discuss the emergent nature of tasks and task constraints, propose the classification of all types of constraints on the basis of their relative rate of change, and hypothesize about their temporally nested organization.

The definition of task constraints as systemic emergent properties of the organism/environment level provides a dimensional reduction of the constraints-led approach. Additionally, as all types of slow-changing constraints subjugate the rapidly changing constraints, a nested and correlated organization of constraints, interacting through circular causality, is hypothesized. The knowledge of such nested organization may help coaches understand and improve the efficiency of learning/training processes.
